# The complete chloroplast genome sequence of *Prunus salicina* ‘Wushan plum’

**DOI:** 10.1080/23802359.2021.1904802

**Published:** 2021-03-26

**Authors:** Bo Fang, Yicen Xu, Jie Yu

**Affiliations:** a Chongqing Academy of Agricultural Sciences, Chongqing, China; b College of Horticulture and Landscape Architecture, Southwest University, Chongqing, China

**Keywords:** *Prunus salicina* ‘Wushan plum’, chloroplast genome, phylogenetic analysis

## Abstract

*Prunus salicina* ‘Wushan plum’ is a local economic fruit crop. In this study, we reported the complete chloroplast genome sequence of *P. salicina* ‘Wushan plum’. The genome has a circular structure of 157,921 bp containing a large single-copy region (LSC) of 86,184 bp, a small copy region (SSC) of 19,031 bp, and two inverted repeats (IR) of 26,353 bp by each. It harbors 110 unique genes, including 78 protein-coding genes, 4 ribosomal RNA genes, and 28 transfer RNA genes. The phylogenomic analysis shows that *P. salicina* ‘Wushan plum’ is clustered with *Prunus salicina*.

*Prunus salicina* 'Wushan plum' is the local characteristic variety of Chongqing. It belongs to *Prunus* of Rosaceae. It is a kind of Chinese plum (*Prunus. salicina* L.). ‘Wushan plum’ has a good yield and strong adaptability. The fruit has good characters in shape and color, fragrant, sour and sweet, and nutritious (Zhao et al. 2019).

*P. salicina* 'Wushan plum' was collected from Wushan, Chongqing. The DNA library was constructed using the Agilent 2100 and sequenced by using the Illumina NovaSeq 6000 sequencing platform.

The chloroplast genome was assembled from the clean data by GetOrganelle (v. 1.6.4) (Jin et al. 2020). The annotation of the chloroplast genome was conducted initially using CpGAVAS2 (Linchun et al. 2019). Furthermore, the annotations with problems were manually edited by using Apollo (Misra and Harris 2006). The genome sequence and annotations have been deposited in the GenBank with accession number MW406458.

The chloroplast genome of *P. salicina* ‘Wushan plum’ is 157,921 bp in size with a large single-copy region (LSC) of 86,184 bp, small copy region (SSC) of 19,031 bp and two inverted repeats (IRs) of 26,353 bp by each. The chloroplast genome of *P. salicina* ‘Wushan plum’ comprises 131 genes, among which, 110 are unique genes, including 78 protein-coding genes, 4 ribosomal RNA (rRNA) genes, and 28 transfer RNA (tRNA) genes. Among the 78 protein-coding genes annotated, nine unique genes contain only one intron, two genes (*ycf*3, *clp*P) contain two introns, and six tRNA genes contain one intron. The length of the protein-coding sequence (CDS) in the chloroplast genome of *P. salicina* ‘Wushan plum’ is 79,728 bp. In contrast, the length of the rRNA genes is 9,048 bp, and the length of the tRNA genes is 2,815 bp. The GC content analysis showed that the overall GC content is 36.72%. Note that the GC contents in IR regions (42.62%) are significantly higher than that in LSC (34.51%) and SSC regions (30.37%).

**Figure 1.**
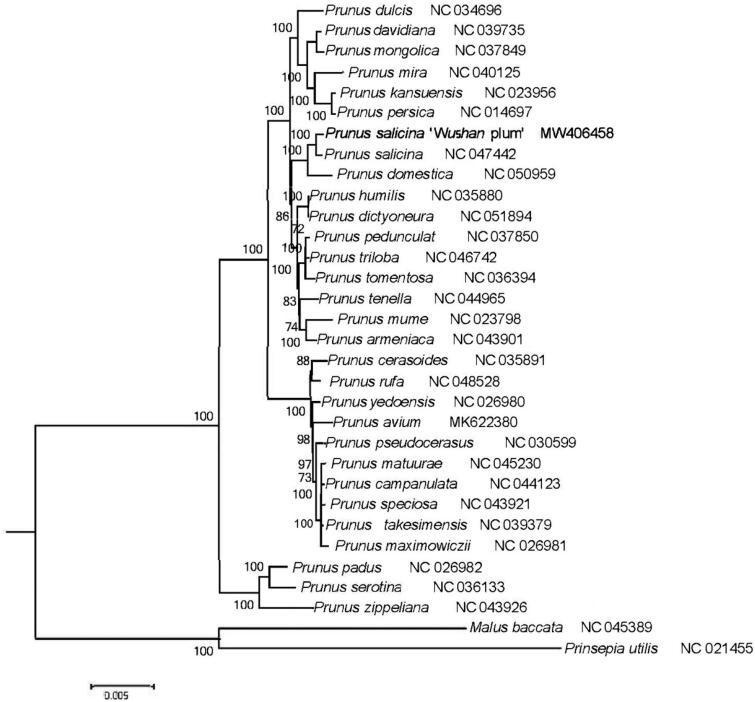
Maximum-likelihood phylogenetic tree for genus *Prunus* L. based on 32 complete chloroplast genomes. Values along branches correspond to ML bootstrap percentages.

To examine the phylogenetic position of *P. salicina* ‘Wushan plum’, we constructed the maximum likelihood (ML) trees by using the *P. salicina* ‘Wushan plum’ and other 29 *Prunus* species and two outgroups complete chloroplast genome sequences. Two species, *Malus baccata* (Rosaceae) and *Prinsepia utilis* (Rosaceae) were selected as outgroups. The complete chloroplast genome sequences were aligned by using MAFFT (https://mafft.cbrc.jp/alignment/server/) online version 7.471 (John et al. 2019). These aligned sequences were used to construct the maximum likelihood tree by RaxML (v8.2.4) (Stamatakis 2014). The phylogenetic analysis showed *P. salicina* ‘Wushan plum’ was clustered with *Prunus salicina* (Figure 1).

## Data Availability

The genome sequence data that support the findings of this study are openly available in GenBank of NCBI at https://www.ncbi.nlm.nih.gov/ under the accession number MW406458. The associated ‘BioProject’, ‘SRA’, and ‘Bio-Sample’ numbers are PRJNA699156, SRR13627323, and SAMN17767268 respectively.
